# Editorial: Genetics of plant-nematode interactions

**DOI:** 10.3389/fpls.2023.1181564

**Published:** 2023-03-16

**Authors:** Isabel Luci Conceição, Carolina Escobar, Charles Opperman, Hendrika Fourie

**Affiliations:** ^1^ University of Coimbra, Department of Life Sciences, Centre for Functional Ecology - Science for People and The Planet (CFE), Coimbra, Portugal; ^2^ Facultad de Ciencias Ambientales y Bioquímica, Universidad de Castilla-La Mancha, Toledo, Spain; ^3^ International Research Organization for Advanced Science and Technology (IROAST), Kumamoto University, Kumamoto, Japan; ^4^ Department of Entomology & Plant Pathology, North Carolina State University, Raleigh, NC, United States; ^5^ Unit for Environmental Sciences and Management, North-West University, Potchefstroom, South Africa

**Keywords:** genetics, plant parasitic nematodes (PPN), interactions, integrated pest and disease management, control strategies

When I was invited to be guest editor of this Research Topic, I was happy and honoured for several reasons. I have never served on the editorial side of the publication process, I love nematodes, and the Research Topic included one of my favourite subjects. Later in this “adventure”, I was joined by three colleagues: Carolina Escobar from Spain, Charles Opperman from the other side of the ocean (USA), and Hendrika Fourie from South Africa. Our objective was to combine some of the most recent and original research on the genetics of Plant-Nematode interactions in this topic, seven papers have been accepted for publication, presenting their research to the scientific community. Science is more than just doing experiments; science is also sharing and knowing how to share the knowledge gained.

Plant-parasitic nematodes (PPN) affect crops worldwide and are one of the main causes of crop damage and crop yield economical loss. As our world seems destined to famine if we cannot improve plant and crop production before 2050, effective and sustainable control of plant pests and diseases becomes of crucial importance. Most current common pest control practices are outdated, and most of the synthetic nematicides are being or are already banned due to their unacceptable impacts on nature and in humans’ health. Novel, less toxic PPN control methods compatible with sustainable agriculture are a priority. In this respect, knowledge of how plants and nematodes interact at the genetic level is of critical importance to identify tools to develop new control strategies and increase production, and thus fight famine and economic losses. In this context, several approaches were used by the authors of the seven articles included in the topic.

Khoei et al. provided a novel insight into the signalling and regulatory network response of soybean (*Glycine max*) to the soybean cyst nematode (*Heterodera glycines*) and *Rotylenchus reniformis*. Their research revealed the involvement of several nematode genes expressed in soybean as a response to the nematodes. The same nematode and crop were also the focus of Lian et al. that consider *H. glycines* as one of the major threats to soybean in China. Several spread patterns of the nematode and the evidence of selection of virulent nematode populations in the field were reported. In addition, the importance of the implementation of a wider crop rotation and planting resistant cultivars for sustainable management of soybean in China were emphasized. These two approaches, although different, reflect the reality of each country and could both be used in the future to develop new strategies against soybean cyst nematode.

*Globodera* spp., another cyst nematode of major importance, was studied. Zheng et al. focused on the gene Gpa2 which confers resistance against *G. pallida*, considered a quarantine species by the European and Mediterranean Plant Protection Organization (EPPO). The data obtained from comparative transcriptomics revealed that resistance against this cyst nematode depends on conserved downstream pathways and is related to the resistance of potatoes against other pathogens. This finding is relevant because knowing the transduction cascades governing plant resistance against this nematode species could help find solutions against other pathogens of great agricultural importance.

Two other articles were dedicated to a new emergent threat to rice (*Oryza sativa*), *Meloidogyne graminicola*. Dash et al. analyzed rice mutants resistant to this root-knot nematode (RKN) and demonstrated by sequencing that the presence of structural genetic variation related to stress resistance and other traits may be involved. Nguyen et al. focused on the rice cultivar Zhonghua 11 resistant to *M. graminicola*. Their results indicated that salicylate signalling and autophagy are activated and may contribute to the resistance-mediated hypersensitive response observed for this emergent RKN. Suzuki et al. described the role of local auxin synthesis during gall formation. Among the auxin biosynthesis enzymes, YUCCA4 (YUC4) was dramatically up-regulated during RKN infection, suggesting it may be a major contributor to the auxin accumulation during gall formation. Although auxin biosynthesis during gall formation comes from multiple sources, one of them is YUC4. The coordination of those auxin sources during gall formation adds more complexity of hormonal regulations during PPN interaction.

Finally, the paper from Hu et al. deals with the pine wilt disease caused by the pinewood nematode (PWN), *Bursaphelenchus xylophilus*, which constitutes one of the most important diseases in world forestry. Spread to the far East and some parts of Europe by world trading, it is causing serious damage to pine forests. The authors identified the pathogen-associated molecular pattern (PAMP) BxCDP1 in *B. xylophilus* and showed that BxCDP1 plays a critical role in the interaction between *B. xylophilus* and *Pinus thunbergia.* They proved that two peptides (M9 and M16) are key BxCDP1 regions to induce PAMP-triggered immunity (PTI) in *Nicotiana benthamiana*; those peptides (M9 and M16) thus have the potential to be developed and used as immune inducers of pines against *B. xylophilus* in the near future.

A deep knowledge of how plants and nematodes interact with each other is essential to tackle control of nematodes and other pests. The plant mechanisms inherent to plant resistance to nematodes are in ways similar to those used against other pests and diseases. Discovering the mechanisms behind those plant-nematode interactions can be an important step to discover how to protect crops from many parasites contributing to the major problem of a predicted future famine. The complexity of this topic implies that its study should be done step by step, until the integration of all parts makes a whole.

## Author contributions

All authors listed have made a substantial, direct, and intellectual contribution to the work and approved it for publication.

